# Supplementary material for: Gamma delta T cells recognize haptens and mount a hapten-specific response
Source: eLife. 2014 Sep 25;3:e03609. doi: 10.7554/eLife.03609 (PMC4174581; doi:10.7554/eLife.03609)
Supplement: Supplementary file 1. — Primers used in this study. DOI: http://dx.doi.org/10.7554/eLife.03609.009 [file elife03609s001.docx]

**Supplementary File 1**

The primers for BioMark qPCR were purchased from Applied Biosystems, including: B2M (Mm00437764_m1), CD3E (Mm00599683_m1), CD19 (Mm00515420_m1), A630038E17RIK (TCRD) (Mm00553757_m1), CD25 (Mm00434261_m1), CD27 (Mm01185212_g1), IL1B (Mm01336189_m1), IL2 (Mm00434256_m1), IL4 (Mm00445260_m1), IL6 (Mm00446191_m1), IL10 (Mm01288386_m1), IL12A (Mm00434169_m1), IL17A (Mm00439619_m1), IL17F (Mm00521423_m1), IL22 (Mm00444241_m1), IL23A (Mm00518984_m1), IFNG (Mm01168134_m1), TNF (Mm00443258_m1), IFNB1 (Mm00439546_s1), TGFB1 (Mm01178820_m1), CCL5 (Mm01302427_m1), CCR2 (Mm00438270_m1), CCR6 (Mm01323931_m1), CCR7 (Mm01301785_m1), GATA3 (Mm00484683_m1), CSF2 (Mm99999059_m1), RORC (Mm01261022_m1), FOXP3 (Mm00475165_m1), BCL6 (Mm00477633_m1), FOS (Mm00487425_m1), CTLA4 (Mm00486849_m1), CXCR4 (Mm01292123_m1), IL1R1 (Mm00434237_m1), IL12RB1 (Mm00434189_m1), IL23R (Mm00519943_m1), AHR (Mm00478932_m1), TLR1 (Mm00446095_m1), TLR2 (Mm00442346_m1), TLR4 (Mm00445273_m1), HPRT1 (Mm00446968_m1), GZMB (Mm00442834_m1), GZMD (Mm01722569_g1), PRF1 (Mm00812512_m1), EOMES (Mm01351985_m1), FASL (Mm00438864_m1), LTA (Mm00440229_g1).
